# Corneal Subbasal Nerve Recovery in an Acute Case of Ultraviolet Keratitis Treated with Autologous Serum Eye Drops

**DOI:** 10.1155/2018/4905487

**Published:** 2018-02-26

**Authors:** Farshad Abedi, Pedram Hamrah

**Affiliations:** ^1^Ocular Surface Imaging Center, Massachusetts Eye and Ear Infirmary, Department of Ophthalmology, Harvard Medical School, Boston, MA 02114, USA; ^2^Cornea Service, Massachusetts Eye and Ear Infirmary, Harvard Medical School, Boston, MA 02114, USA; ^3^Boston Image Reading Center, Department of Ophthalmology, Tufts Medical Center, Tufts University School of Medicine, Boston, MA, USA; ^4^Cornea Service, New England Eye Center, Department of Ophthalmology, Tufts Medical Center, Tufts University School of Medicine, Boston, MA, USA

## Abstract

**Purpose:**

To report degeneration of subbasal corneal nerves and the subsequent neuroregeneration in a case of acute ultraviolet (UV) keratitis, treated with autologous serum eye drops.

**Methods:**

Case report.

**Results:**

A 37-year-old female presented with ocular discomfort and blurred vision in both eyes, after exposure to UV-C light in a laboratory. On exam, she had bilateral conjunctival injection and superficial punctate keratitis (SPK), worse in the left, consistent with acute, bilateral, but asymmetric UV-C keratitis. She was initially started on antibiotic ointment and lubricant eye drops. On her follow-up visit 3 days later, corneas had persistent SPK bilaterally. Laser scanning in vivo confocal microscopy (IVCM) showed beading of subbasal corneal nerves in the right eye and decreased subbasal corneal nerve density and numerous amputated nerves in the left eye. Autologous serum eye drops 20%, eight times a day, and loteprednol 0.5% ophthalmic solution were commenced in both eyes. Twelve weeks later, her symptoms fully resolved; IVCM revealed near-normal subbasal corneal nerve density in both eyes.

**Conclusions:**

IVCM demonstrated dramatic damage to subbasal corneal nerves after brief UV-C exposure. The patient, treated with autologous serum eye drops in both eyes, achieved resolution of symptoms and recovery of subbasal corneal nerves.

## 1. Introduction

Ultraviolet (UV) keratitis is characterized by severe ocular pain and decreased visual acuity (VA) six to twelve hours after a relatively short exposure to light sources [1]. Slit-lamp examination shows typically bilateral superficial punctate keratitis (SPK). In severe cases, this can be followed by total epithelial defects [1]. Corneal reepithelialization, aided by lubricating eye drops, bandage contact lenses, or patching of the eyes, usually occurs over a 36- to 72-hour period, and long-term sequelae are rare [1]. Recently, however, we showed that UV exposure may result in corneal neuropathy-induced photoallodynia [2]. Acosta et al. have demonstrated that sensitization of nociceptors and depression of cold thermoreceptor activity can occur in UV-induced keratitis in guinea pigs [3]. They suggested that changes in nerve activity possibly underlie the sensation of discomfort and pain, which are associated with ocular surface inflammation induced by UV exposure.

Laser in vivo confocal microscopy (IVCM) is a noninvasive, high-resolution device that allows imaging of the living cornea at the cellular level, providing images comparable with histochemical methods. In recent years, the use of IVCM has revealed the importance of corneal nerves in healthy eyes and ocular diseases [4, 5]. Using laser IVCM, we have recently demonstrated a dramatic decrease in subbasal corneal nerves in patients with various types of microbial keratitis and in patients with corneal neuropathy-induced photoallodynia [2, 4, 6–8]. Herein, we report, for the first time, the alterations and acute degeneration of subbasal corneal nerves, followed by complete subbasal corneal nerve regeneration and resolution of symptoms in a patient with acute UV keratitis, treated with autologous serum eye drops.

## 2. Case Report

A 37-year-old female presented to the emergency room with severe pain, blurry vision, photophobia, and redness in both eyes, worse in the left eye. Her symptoms started about five hours after she was exposed for approximately 5–10 minutes to UV-C light that was not turned off in a laboratory culture hood. Her past ocular history was remarkable for anisometropia and congenital corectopia in the left eye. She only wore contact lenses in her right eye. Her past medical history was otherwise unremarkable, and she was not taking any medications. At the time of presentation, her visual acuity (VA) in the right eye was counting fingers (CF) at 4 feet, unaided, that improved to 20/125 with pinhole correction. Her VA in the left eye was 20/70, unaided, with no improvement with pinhole correction. On slit-lamp examination, her right eye demonstrated mild conjunctival injection and a diffuse confluent superficial punctate keratitis (SPK) with no limbal ischemia. Pupil, anterior chamber (AC), lens, and retinal examinations were unremarkable. Her left eye revealed corectopia, with a superiorly displaced oval pupil, moderate conjunctival injection, diffuse dense confluent SPK, a nasal posterior embryotoxon with no limbal ischemia, and a deep AC. Lens and retinal examination findings were within normal limits.

**Figure 1:**
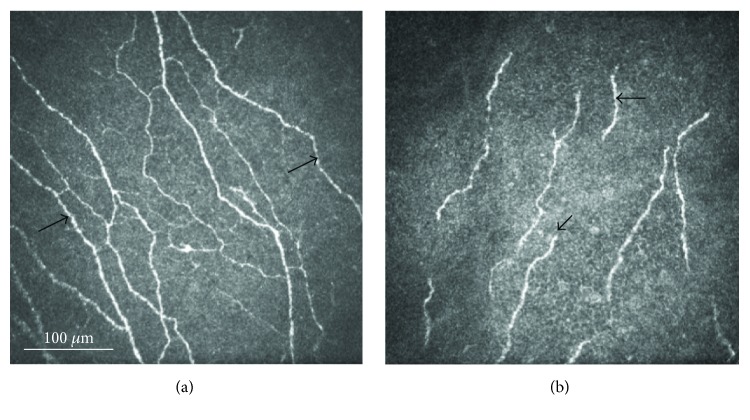
In vivo confocal microscopy images of both eyes three days after exposure to ultraviolet-C light demonstrating nerve beading (arrows) of the right eye (a) and decreased nerve density with fractured nerve pieces and amputated subbasal corneal nerves (arrows) in the left eye (b).

She was diagnosed with bilateral UV-C keratitis, more severe in the left eye, and initially treated with bacitracin/polymyxin B ophthalmic ointment in both eyes, six times a day, cyclopentolate 1% three times a day in the left eye, chilled artificial tears, and cold compresses in both eyes as required. On her follow-up visit at the cornea service 3 days later, her symptoms had slightly improved. Uncorrected VA had increased to 20/400 in the right eye, 20/60 with pinhole, and 20/40 in the left eye. Her conjunctiva injection had improved, but corneas showed persistent SPK in both eyes. Laser IVCM (Heidelberg Retina Tomograph 3/Rostock Cornea Module, Heidelberg Engineering GmbH, Heidelberg, Germany) of the central cornea was performed and showed beading of the subbasal corneal nerves in the right eye and deceased nerve density with fractured and amputated subbasal corneal nerves in the left eye (Figure 1). Quantitative analysis of subbasal nerves was carried out using the semiautomated tracing program NeuronJ, a plug-in for ImageJ (available at http://www.imagescience.org/meijering/software). Nerve density was assessed by measuring the total length of the subbasal nerve fibers in the central cornea. Total nerve density was 24.9 mm/mm^2^ in the right eye, which is within normal range, [4, 8] but only 9.1 mm/mm^2^ in the left eye.

**Figure 2:**
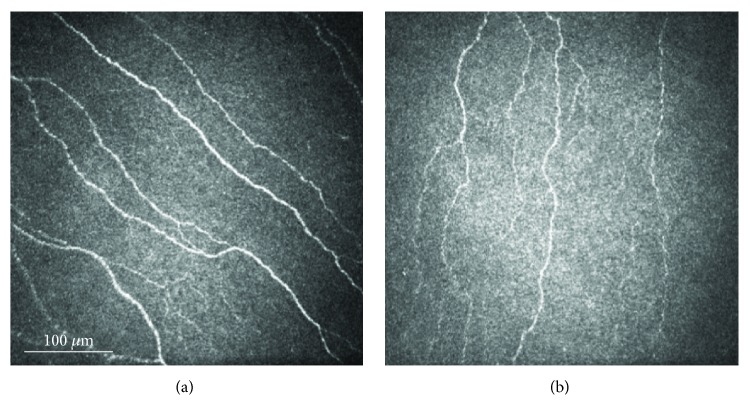
In vivo confocal microscopy images of both eyes after a twelve-week treatment with autologous serum eye drops showing resolution of nerve beading in the right eye (a) and increased nerve density in the left eye (b).

Autologous serum eye drops diluted at 20%, eight times a day, and a taper of loteprednol 0.5% ophthalmic solution, one drop four times a day for two weeks, then two times a day for two weeks, were commenced in both eyes. In addition to acute management, the rationale for this treatment was also to prevent long-term sequelae, such as corneal neuropathic disease [2]. On her next follow-up visit four weeks later, her symptoms had significantly improved while she was at home, but she reported dryness, grittiness, and light sensitivity when outdoors. Her uncorrected VA had improved to 20/30 (20/25 with pinhole) in the right eye and 20/40 (20/30 with pinhole) in the left eye. IVCM revealed increased subbasal corneal nerves in both eyes. She was advised to continue autologous serum eye drops, eight times a day, and decrease loteprednol 0.5% ophthalmic solution, to one drop per day in both eyes. She was followed up again 8 weeks later. At that time, her symptoms had fully resolved and slit-lamp examination showed white and quiet conjunctiva and resolved corneal SPK in both eyes. Repeat laser IVCM demonstrated decreased beading of subbasal corneal nerves with a total nerve density of 21.6 mm/mm^2^ in the right eye and nearly doubling to 16.9 mm/mm^2^ in the left eye (Figure 2).

## 3. Discussion

Herein, we demonstrate, for the first time, active corneal nerve damage including amputated subbasal corneal nerves within 3 days after exposure to UV-C light. In this patient, the symptoms and signs were more severe in her left eye than those in the right eye, potentially due to a protective effect of the contact lens worn in the right eye. Subbasal corneal nerve densities were decreased only in the left eye compared with reference normal controls [8]. This report may suggest that treatment with autologous serum eye drops could result in faster resolution of signs and symptoms of UV-related keratopathy and regeneration of subbasal corneal nerves.

The limitation of this case report is that we were not able to assess to natural response and clinical signs and symptoms to extensive UV-C exposure without intervention. In addition, we were not able to assess if corneal nerve would regenerate without autologous serum tears in this case and if they do at what rate. In our case, with autologous serum eye drops, subbasal nerve density improved from 9.1 mm/mm^2^ to 16.9 mm/mm^2^ over 12 weeks in the left eye. Compared to a recent report, in which we demonstrated subbasal corneal regeneration rate of 1.02 ± 0.52 mm/mm^2^ per month in patients with infectious keratitis [8], we observed that the subbasal corneal nerves in our case regenerated at a rate of 2.6 mm/mm^2^ with the use of autologous serum drops, which was much more rapid.

Corneal nerves typically release neuronal factors that promote corneal epithelial homeostasis and play a role in maintaining the functional integrity of the ocular surface. The use of IVCM has recently contributed to our knowledge on the importance of corneal nerves in ocular diseases, such as in infectious keratitis, neurotrophic keratopathy, and dry eye disease [2, 8, 9]. Loss of corneal nerves has previously been demonstrated in patients with dry eye disease, microbial keratitis, and in patients with corneal neuropathy [2, 8–10]. In contrast, we recently demonstrated that corneal nerve regeneration may be induced with autologous serum eye drop treatment and resulted in improvement of patients' symptoms such as photoallodynia [2]. Another study reported significant increase in mean number, length, width, and density of subepithelial nerves after treatment with autologous plasma therapy in the eyes with neurotrophic keratopathy [11].

Clinically, a recent randomized clinical trial compared a 2-week treatment with topical 20% autologous eye drops to conventional artificial tears in patients with severe dry eye syndrome [12]. Autologous serum treatment resulted in a statistically significant improvement in symptoms compared with conventional artificial tears using the standardized ocular surface disease index (OSDI). Another prospective randomized crossover study also reported a significantly greater decrease in OSDI score and a higher TBUT in the autologous serum treatment group after 1 month of treatment, compared with conventional preservative-free artificial tears [13]. These findings are consistent with our outcomes using autologous serum eye drops in the present case report with UV keratitis. Autologous serum eye drops contain several growth factors, including nerve growth factor, insulin-like growth factor-1, brain-derived neurotrophic factor, neurotrophin-3, and glial cell line-derived neurotrophic factor [14]. These growth factors may play a role in nerve regeneration and result in the improvement of ocular surface disease in these patients. Further research on the changes in corneal nerves in ocular surface diseases, using laser IVCM, can lead to a better understanding of the role of corneal nerves in heathy eyes and different ocular surface diseases.
